# Valuable carcasses: postmortem preservation of fatty acid composition in heart tissue

**DOI:** 10.1093/conphys/coz005

**Published:** 2019-02-20

**Authors:** Shannon E Currie, Laurent Mène-Saffrané, Nicolas J Fasel

**Affiliations:** 1 Department of Evolutionary Ecology, Leibniz Institute for Zoo and Wildlife Research, Alfred Kowalke Str. 17, Berlin, Germany; 2 Department of Biology, University of Fribourg, Chemin du Musée 10, Fribourg, Switzerland; 3 Metabolomics and Proteomics Platform, University of Fribourg, Chemin du Musée 10, Fribourg, Switzerland

**Keywords:** Bat, *Carollia perspicillata*, ethics, euthanasia, fatty acid, heart

## Abstract

In order to effectively conserve species, we must understand the structure and function of integral mechanisms at all levels of organismal organisation, from intracellular biochemistry to whole animal ecophysiology. The accuracy of biochemical analyses depend on the quality and integrity of the samples analysed. It is believed that tissue samples collected immediately postmortem provide the most reliable depiction of the living animal. Yet, euthanasia of threatened or protected species for the collection of tissue presents a number of ethical complications. Polyunsaturated fatty acids (PUFA) are essential to the cardiovascular system of all animals and the structure of PUFA can be degraded by peroxidation, potentially modifying the fatty acid composition of the tissue over postmortem time. Here, we assessed the composition of PUFA in cardiac tissue of bats (*Carollia perspicillata*) over the course of 12-h postmortem. We show that PUFA are resistant to naturally occurring postmortem degradation in heart tissue, with no difference in the overall composition of fatty acids across all time classes (0, 3, 6 or 12-h postmortem). Our results suggest that carcasses that would otherwise be discarded may actually be viable for the assessment of fatty acid composition in a number of tissues. We hope to spur further investigations into the viability of carcasses for other biochemical analyses as they may be an untapped resource available to biologists.

## Teaser

Tissue degradation postmortem is an issue for the collection of representative biochemical data. We show that, for heart tissue, fatty acid composition is preserved up to 12-h postmortem. We advocate that carcasses may be a valuable resource in cases where euthanasia should be avoided because of ethical or conservation consideration.

## Introduction

A comprehensive understanding of physiological functions requires the direct sampling of relevant tissues from an animal. Unfortunately, tissue samples are generally difficult to obtain without animal euthanasia. While biopsy techniques are improving, there are still organs for which this is not viable, especially for small animals. Current ideology dictates that tissue samples collected immediately postmortem provide the most accurate representation of the living organism. Recently, researchers have begun investigating the functionality of intracellular process such as mitochondrial ATP production in relation to postmortem time (Barksdale *et al.*, 2010); as well as the viability of cadaver tissue for use in transplantation and regenerative medicine (O’Driscoll *et al.*, 1999; Shikh Alsook *et al.*, 2015). The results of these studies suggest that cadaver tissues may remain viable anywhere from 24-h postmortem (Sun *et al.*, 2016, Káš *et al.*, 1969) to up to 17 days (Latil *et al.*, 2012). To our knowledge only one other study has been conducted on wildlife models (Guinea pigs; Kas *et al.* 1969) to investigate the potential use of tissues collected many hours after death for animal physiological studies.

While comparative species sampling (i.e mice/pigs as a proxy for humans) may provide insight into broadly analogous tissue function, direct samples from the species of interest are undoubtedly preferred. In threatened and protected species, the ethical considerations and justification for euthanasia solely for tissue collection is exceedingly complicated. Yet, these species are often those for which biochemical and physiological data are lacking. In order to effectively conserve species, the understanding of structural and functional mechanisms taking place at all levels of organismal organisation must be acquired. Most importantly, we need information regarding the composition of macromolecules required for adequate function in the major physiological systems such as immunity, reproduction, digestion and circulation.

Polyunsaturated fatty acids (PUFA) are essential to vertebrates as they are not produced *de novo* and consequently must be acquired through the diet. These fatty acids also have low melting temperatures and enhance cellular membrane fluidity. In mammals, PUFA are crucial for supporting the cardiovascular system (Anderson and Taylor, 2012). This is particularly true for species exhibiting profound changes in metabolism such as daily heterotherms and hibernators (Giroud *et al.*, 2013). The characteristics and importance of PUFA have been widely studied in a number of physiological systems and continue to interest biologists from a range of backgrounds (Arnold *et al.*, 2015; Esmaeili *et al.*, 2015).

Bats in particular experience immense fluctuations in metabolism and cardiovascular function over short time frames with changes in heart rate from 5 bpm during torpor, to upwards of 1000 bpm during flight (Studier and Howell, 1969; Currie *et al.*, 2018). Yet, PUFA composition as it pertains to the cardiovascular system of bats remains understudied. Bats are elusive mammals which are active at night and often fly at high altitudes where capturing them becomes difficult. In addition, a large number of bat species are vulnerable to disturbance with populations declining globally (Mickleburgh *et al.*, 1992; Hutson *et al.*, 2001) and recent species extinctions (e.g. Martin *et al.*, 2012). Therefore, collection and euthanasia of individuals for tissue sampling can be problematic due to necessary protection legislation.

Here, we investigated the viability of tissue samples taken from bat carcasses up to 12h postmortem compared with those taken from freshly euthanized individuals. As more and more animals are being kept in zoos and wildlife refuges, we felt it important to investigate the viability of carcasses collected in these institutions as they may provide a valuable resource to physiologists. This timeframe was selected as the carcasses animals in most captive conditions are likely to be discovered within this timeframe. We specifically focused on the composition of fatty acids in a vital organ for which alternatives and biopsy are often not viable options- the heart. We predicted that there would be a degradation of PUFAs by 12-h postmortem, and hypothesised that n-3 fatty acids would show a greater level of degradation than n-6 fatty acids, because of their greater sensitivity to lipid peroxidation (Hulbert *et al.*, 2007).

## Methods

### Tissue collection

We used carcasses of 19 adult *Carollia perspicillata* (7 female, 12 male) chosen at random from a captive population located in a tropical zoo (Papillorama, Kerzers, Switzerland). Culling was independent from the present study. Individuals were euthanized in the morning (~9:00 am) via an intraperitoneal overdose of sodium pentobarbital and then selected at random for inclusion in our study. Following euthanasia, bat carcasses were kept whole in perforated plastic bags at room temperature (25 ± 1°C). We randomly assigned individual carcasses to four treatment groups based on time since death, with four bats per time treatment (0, 3, 6 or 12-h postmortem). As we were interested in the effect of postmortem time on carcass viability, we felt it most appropriate to assess whole hearts from intact individuals rather than biopsy a single dissected heart over the course of 1 day. Following the allotted time treatment, we dissected the whole heart from the body and placed it in a plastic vial which was then flash frozen using liquid nitrogen. It took no longer than 30 min to perform all five dissections per treatment group.

### Phospholipid extraction

Lipids were extracted using an established protocol (Bligh and Dyer, 1959). Approximately 100 mg of tissue was crushed in liquid nitrogen and put in a 2-ml tube with 290 μl of MeOH, 150 μl of CHCl_3_ and 10 μl of butylated hydroxytoluene (BHT, antioxidant, 2 mg/ml) diluted in MeOH (0.1% w/v MeOH). The suspended tissue was then shaken for 15 min at 10Hz. Following the addition of 300 μl of H_2_O and 150 μl of CHCl_3_ the suspended tissues were then vigorously shaken for 5 min before being centrifuged for a further 5 min at 3500 *g*. Finally, 250 μl of the lower suspension phase was collected and phospholipids were separated through solid phase extraction. In 7-ml glass columns, 500 mg of silica powder was compacted between two PTFE frits. After conditioning the column with 5 ml of CHCl_3_, the lipid extract was loaded into the silica powder. Subsequently, 5 ml of CHCl_3_ and 2 × 5 ml of acetone were loaded on the column and left to drain. After the second load of acetone, the column was vacuum dried. Finally, the phospholipids were eluted from the column using 5 ml of MeOH, and samples were evaporated under a nitrogen flux prior to resuspension in 50 μl of CHCl_3_.

### Fatty acid analysis

Fatty acid methyl esters (FAMEs) were prepared by incubating the 50-μl samples with 1-ml of 5% (v/v) H_2_SO_4_ in MeOH and BHT (0.1%, w/v). We performed a transesterification reaction in a dry block heater (VWR, Dietikon, Switzerland) set at 85°C for 30 min. At the end of the reaction, tubes were cooled down to room temperature, briefly spun down, and 1.5 ml of 0.9% (w/v) NaCl and 2 ml of *n*-hexane were added to the solution. The mixture was then shaken vigorously for 5 min and both organic and hydroalcoholic phases were separated by centrifugation at 1500*g* for 5 min. The upper organic (*n*-hexane) phase was transferred into a new glass tube. The *n*-hexane phases were evaporated under a nitrogen flux and FAMEs were re-suspended in 30μl of *n*-hexane. We then transferred FAME samples into 0.2-ml crimp vials (BGB Analytik, Genève, Switzerland) prior to injection in the gas chromatography (GC).

We used a GC coupled to a flame ionization detector (Agilent 7890A) to analyse FAME samples. The samples were introduced to the injection port heated at 250°C with an automated-liquid sampler (Agilent 7993) and were split 100 times before injection. FAMEs were separated on 30-m long × 0.25-mm ID × 0.25 μm DB-23 capillary column (Agilent) using He as vector gas (2.6 ml/min). The oven temperature, initially set at 100°C for 2 min, was first increased to 160°C at 25°C/min, then to 250°C at 8°C/min and maintained at this temperature for an additional 4 min. We set the detector temperature to 270°C, while detector gases were set at 30 ml/min for H_2_, 400 ml/min for air and 30 ml/min for makeup gas (He). Data were recorded at a frequency of 50 Hz. We then performed FAME quantifications with calibration curves built with the Supelco 37 component FAME mix (Sigma) using 17:0 methyl ester as the internal standard (250ng of Glyceryl triheptadecanoate). FAME determination was based on the retention time of each component and compared with a Supelco 37 FAME mix (Sigma).

### Statistical analyses

The different types of FAMEs were classified as n-6 PUFAs, n-3 PUFAs, monounsaturated (MUFAs) or saturated fatty acids (SFAs). The proportion of each fatty acids class over the total amount of FAMEs measured were calculated. We conducted all analyses in R (version 3.3.2) and the significance level was set at 5%.

In order to evaluate potential changes in the various fatty acid over time, we ran Type II MANOVAs (function lm and ANOVA) with time since death considered as an explanatory variable. As the response variables (i.e. the fatty acid classes) are interdependent, we considered them as closed composition (function: acomp, package: composition; Van den Boogart *et al.*, 2014). In order to fulfil the assumption of normality, we further transformed them with isometric log-ratio (function ilr, package: composition). In addition, we analysed the effect of time on each fatty acid classes with univariate linear models (function lm). The response variable was logit-transformed to fit tests’ assumptions.

## Results

**Figure 1:**
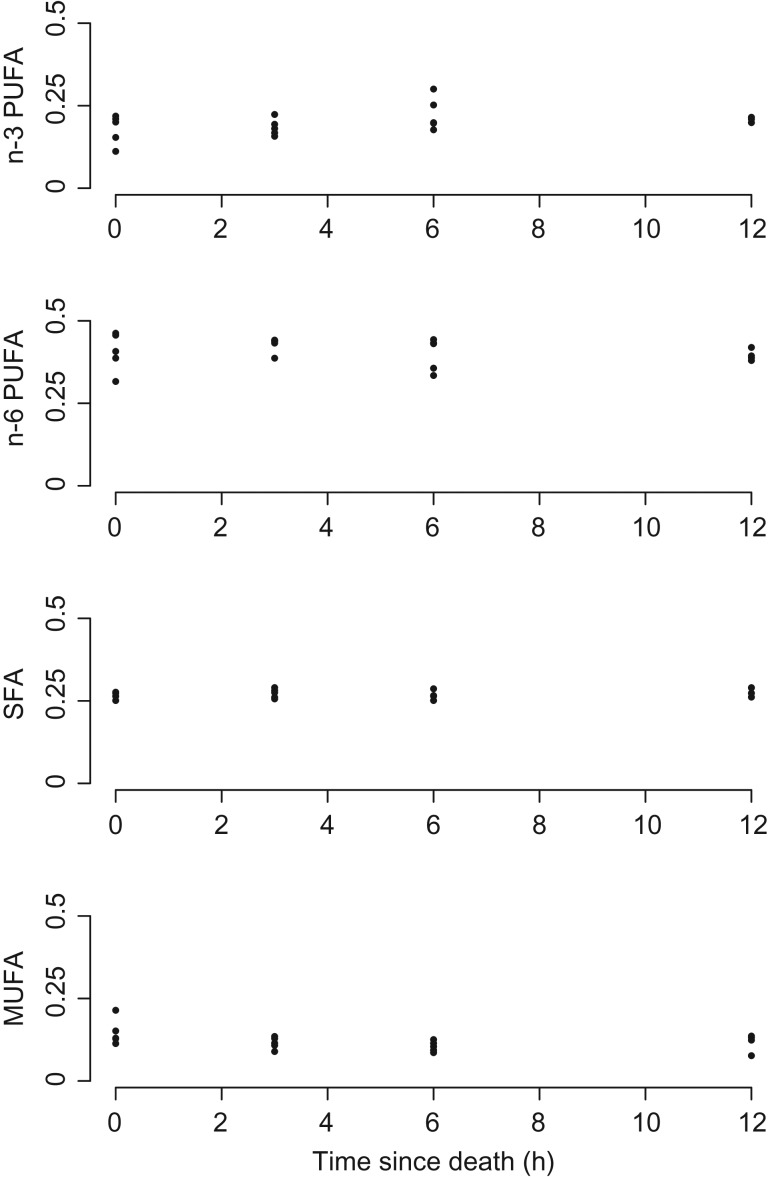
Proportion of different fatty acid classes measured at different times since death in heart of *Carollia perspicillata*

Immediately postmortem the fatty acid composition of *C. perspicillata* hearts was made up of ~58% PUFA compared with ~27% SFA and ~15% MUFA (Table 1). The proportional composition did not vary significantly with time since death as the hearts collected at 12h consisted of ~60% PUFA, ~28% SFA and ~12% MUFA (Table 1). This was supported by the results of MANOVA analysis with no significant change in fatty acid composition across all time classes following the death of the animal (Fig. 1, Pillai test statistic: approx. *F*-value_2,16_ = 1.096, *P* = 0.358). Analysed singly, none of the fatty acid classes changed significantly over the experimental time (Table 2).

**Table 1:** Means and standard deviations of the weight percentage of the different fatty acid methyl esters (FAMEs) and of the various fatty acid classes over the total amount of FAMEs, present in the heart phospholipids of *C. perspicillata*

	Time since death (h)			
	0-h (*n* = 5)	3-h (*n* = 5)	6-h (*n* = 5)	12-h (*n* = 4)
16:0	8.51 ± 0.43	8.69 ± 1.60	8.23 ± 0.96	9.72 ± 1.32
18:0	18.29 ± 1.39	18.59 ± 1.21	18.87 ± 0.85	18.15 ± 1.30
18:1 n-9	9.77 ± 1.12	8.93 ± 0.53	9.39 ± 0.97	8.68 ± 1.34
18:1 n-11	0.37 ± 0.52	0.42 ± 0.58	0.31 ± 0.69	0.94 ± 0.68
18:2 n-6	11.74 ± 3.24	12.31 ± 1.99	12.21 ± 1.84	10.29 ± 1.62
18:3 n-3	0.00 ± 0.00	0.00 ± 0.00	0.00 ± 0.00	0.29 ± 0.58
20:3 n-6	1.96 ± 1.80	3.43 ± 0.36	3.03 ± 0.35	3.36 ± 0.29
20:3 n-3	0.66 ± 1.48	0.00 ± 0.00	0.00 ± 0.00	0.41 ± 0.82
20:4 n-6	20.77 ± 3.85	21.56 ± 2.20	20.32 ± 3.12	20.83 ± 2.20
22:1	1.08 ± 1.90	0.20 ± 0.44	0.00 ± 0.00	0.47 ± 0.94
22:4 n-6	4.69 ± 3.43	3.19 ± 1.05	1.84 ± 1.23	2.87 ± 1.16
22:5 n-6	1.42 ± 1.40	2.26 ± 1.35	2.54 ± 0.78	2.16 ± 0.50
22:5 n-3	1.97 ± 1.11	2.41 ± 0.27	2.79 ± 0.23	3.23 ± 0.59
22:6 n-3	15.05 ± 2.92	16.05 ± 2.52	19.71 ± 5.03	16.99 ± 1.16
24:1	3.53 ± 3.21	1.95 ± 1.40	0.75 ± 1.15	1.62 ± 1.48
PUFA	58.46 ± 3.14	61.21 ± 1.54	62.44 ± 1.04	60.43 ± 2.27
SFA	26.80 ± 1.05	27.28 ± 1.43	27.10 ± 1.55	27.86 ± 1.39
MUFA	14.74 ± 3.98	11.51 ± 1.80	10.46 ± 1.59	11.70 ± 2.74
n-6	40.58 ± 5.95	42.76 ± 2.31	39.94 ± 5.02	39.51 ± 1.71
n-3	17.88 ± 4.51	18.45 ± 2.57	22.50 ± 5.06	20.92 ± 0.75

Animal were stored at room temperature (˜25°C) and heart tissues were collected at various time points after death.

**Table 2:** Effects of time postmortem (0, 3, 6 and 12-h) on proportions of heart fatty acid classes (logit-transformed) in *Carollia perspicillata*

	df	Estimate ± SE	*t*-value	*P*-value
n-6 PUFA	1,17			
Intercept		−0.35 ± 0.06	−5.65	<0.001
Time		−0.01 ± 0.01	−0.66	0.519
n-3 PUFA	1,17			
Intercept		−1.52 ± 0.09	−17.78	<0.001
Time		0.02 ± 0.01	1.63	0.121
MUFA	1,17			
Intercept		−1.91 ± 0.09	−21.42	<0.001
Time		−0.02 ± 0.01	−1.49	0.154
SFA	1,17			
Intercept		−1.00 ± 0.02	−43.93	<0.001
Time		0.00 ± 0.00	1.13	0.276

## Discussion

Here, we present data for fatty acid composition in the hearts of frugivorous bats. Our results show that the fatty acid composition of the heart cellular membrane in *C. perspicillata* is one of the richest in PUFA among species investigated so far (Aloia and Pengelley, 1979; Pamplona *et al.*, 2000; Hulbert *et al.*, 2002). We also highlight that the fatty acid composition of the heart remains stable up to 12-h postmortem. The hearts of heterothermic animals commonly have high PUFA to SFA ratios, particularly n-6 fatty acids, which are thought to support cardiac function at reduced body temperatures. *C. perspicillata* is a daily heterotherm capable of reducing its body temperature to ~20°C during torpor (Audet and Thomas, 1997). In comparison to another heterotherm, the hibernating golden mantled ground-squirrel (*Citellus lateralis*), *C. perspicillata* still showed a greater proportion of PUFA in heart phospholipids ~58% compared with ~51% (Aloia and Pengelley, 1979). This high proportion in PUFA is likely protected against peroxidation by a strong dietary antioxidant capacity, as oxidative damages remain low in frugivorous bat species, like *C. perspicillata* (Wilhelm Filho *et al.*, 2007; Schneeberger *et al.*, 2014; Fasel *et al.*, 2017).

We are currently living in the era of human impact known as the ‘Anthropocene’, where considerable habitat degradation and wildlife population declines have restricted many species to entirely captive populations (Lees and Wilcken, 2009). The management of wildlife has also increased the number of individuals being processed through rehabilitation centers and requiring veterinary interventions (Molony *et al.*, 2006; Grogan and Kelly, 2013). These centers are incapable of ensuring the survival of every individual that comes into their care and thus often have access to relatively fresh carcasses. It has therefore become essential for locations with captive animals to assert management protocols with relation to the use and or disposal of any carcasses. At these facilities, it is common practice to cold store carcasses for extended periods to ensure the maintenance of good records. In addition, the principle of complete utilisation of animal carcasses has been incorporated into the guidelines of a number of zoological organisations worldwide. For example, the American Association of Zoo Veterinarians explicitly states that *‘*A reasonable effort should be made to distribute postmortem specimens to institutions for further research or for museum exhibition’ (Carpenter *et al.*, 2011). However due to the lack of investigations into the viability of tissues collected from carcasses, many remain unused. Our data suggest that these cold stored carcasses may be an important, yet unused source.

The US National Research Council recommends that captive endothermic animals must be housed at temperatures below the lower critical temperature of their thermoneutral zone (National Research Council, 2011), generally below 30°C so as to reduce the risk of heat stress. As there can be many animals kept in any one facility, temperatures are often maintained at an intermediate value around 20°C (National Research Council, 2011; Mellor *et al.*, 2015). It is possible that the exposure of carcasses to higher ambient temperatures could result in swifter degradation of tissues and peroxidation, shortening the time that they remain viable. We show that even at a relatively warm temperature (25°C), cardiac tissue of small bats was still reliable for many hours. While the exact time of death may not always be available in all facilities, in many cases it is likely that animals are found within 12-h postmortem, and we suggest that their tissues may still be useful.

Here, we show that at least for PUFAs in heart tissue, the remains of individuals found up to 12-h postmortem provide a reliable depiction of the living animal. Our study has implications for ethical scientific practice and we advocate that utilising carcasses rather than sacrificing new individuals for each experiment will enable investigators to make use of a pre-existing resource for their research. We encourage the comparison of cadaver tissue in other organ systems to freshly dead animals in hopes of broadening the impact of our findings. The use of cadaver tissue will reduce the need for animal euthanasia and potentially enable biologists to study species for which very little information is available. We believe that wildlife refuge/rehabilitation centers and even zoos represent an essentially untapped resource of tissue samples available to biologists.
